# BrdU-positive cells in the neonatal mouse hippocampus following hypoxic-ischemic brain injury

**DOI:** 10.1186/1471-2202-6-15

**Published:** 2005-03-02

**Authors:** John Bartley, Thomas Soltau, Hereward Wimborne, Sunjun Kim, Angeline Martin-Studdard, David Hess, William Hill, Jennifer Waller, James Carroll

**Affiliations:** 1 The Department of Pediatrics, The Medical College of Georgia, Augusta, GA 30912, USA; 2 The Department of Neurology, The Medical College of Georgia, Augusta, GA 30912, USA; 3 The Department of Cellular Biology and Anatomy, The Medical College of Georgia, Augusta, GA 30912, USA; 4 The Department of Biostatistics, The Medical College of Georgia, Augusta, GA 30912, USA

## Abstract

**Background:**

Mechanisms that affect recovery from fetal and neonatal hypoxic-ischemic (H-I) brain injury have not been fully elucidated. The incidence of intrapartum asphyxia is approximately 2.5%, but the occurrence of adverse clinical outcome is much lower. One of the factors which may account for this relatively good outcome is the process of neurogenesis, which has been described in adult animals. We used a neonatal mouse model to assess new cells in the hippocampus after H-I injury.

**Results:**

Neonatal mice underwent permanent unilateral carotid ligation on the seventh postnatal day followed by exposure to 8% hypoxia for 75 minutes. The presence of new cells was determined by bromodeoxyuridine (BrdU) incorporation into cells with sacrifice of the animals at intervals. Brain sections were stained for BrdU in combination with neuronal, glial, endothelial and microglial stains.

We found a significant increase in BrdU-positive cells in the neonatal mouse hippocampus in the injured area compared to the non-injured area, most prominent in the dentate gyrus (DG) (154.5 ± 59.6 v. 92.9 ± 32.7 at 3 days after injury; 68.9 ± 23.4 v. 52.4 ± 17.1 at 35 days after injury, p < 0.0011). Among the cells which showed differentiation, those which were stained as either microglial or endothelial cells showed a peak increase at three days after the injury in the DG, injured versus non-injured side (30.5 ± 17.8 v. 2.7 ± 2.6, p < 0.0002). As in the adult animal, neurogenesis was significantly increased in the DG with injury (15.0 ± 4.6 v. 5.2 ± 1.6 at 35 days after injury, p < 0.0002), and this increase was subsequent to the appearance of the other dividing cells. Numbers of new oligodendrocytes were significantly higher in the DG on the non-injured side (7.0 ± 24.2 v. 0.1 ± 0.3, p < 0.0002), suggesting that oligodendrocyte synthesis was reduced in the injured hippocampus.

**Conclusion:**

These findings demonstrate that the neonatal animal responds to brain injury with neurogenesis, much like the adult animal. In addition, H-I insult leads to more neurogenesis than hypoxia alone. This process may play a role in the recovery of the neonatal animal from H-I insult, and if so, enhancement of the process may improve recovery.

## Background

The incidence of intrapartum asphyxia was recently determined to be 2.5% in a large population of singleton pregnancies [1]. Even so, the occurrence of adverse neurological outcome following delivery is actually much lower. There are undoubtedly a number of reasons why greater numbers of infants do not suffer lasting neurological impairment, but one of the reasons may be the formation of new cells in the damaged tissue. Recent studies have demonstrated the presence of multipotent neural stem cells in the subventricular zone and the subgranular zone of the hippocampus [2]. Hippocampal stem cells from the adult brain have been demonstrated to proliferate and differentiate into neurons, astrocytes, and oligodendrocytes [3], as a response to multiple factors, including hypoxic-ischemic (H-I) injury, or trauma [4,5].

New neuronal growth has been demonstrated in several studies of adult H-I models, including in humans [6-10], but this finding has received less attention in neonatal animals. The pluripotent stem cells found in the neonatal brain have not been characterized as completely as those in the adult brain. As this process may be relevant to the recovery of the neonatal brain from H-I injury, we sought to determine whether new cells arose in the neonatal brain following this type of injury. Bromodeoxyuridine (BrdU) is a thymidine analog incorporated into the DNA of dividing cells, rendering them detectable by immunohistochemical means [11]. We employed a neonatal mouse H-I brain injury model with BrdU injection to determine what types of BrdU-positive cells are present in the hippocampus after an H-I event and to determine if this response if different than that due to hypoxia alone.

## Results

Figure 2 shows the area of damage produced in the affected hippocampus. The majority of the BrdU positive/NeuN positive cells were found in the granule cell layer on the experimental side. The side of the hippocampus contralateral to ligation, although also exposed to hypoxia, did not demonstrate the same degree of injury, remaining histologically normal in appearance. The finding that common carotid artery occlusion or hypoxia alone does not cause apparent brain damage has been previously reported [12,13]. Thus, we compared the hypoxia alone side of the brain to the hypoxic-ischemic side of the brain in the statistical analyses.

### Regional differences

The DG on the hypoxic-ischemic side of the brain exhibited significantly higher numbers of BrdU-positive cells at P10, P17, and P42 than either CA1 or CA3. BrdU/RCA-positive cells were significantly higher at P10 in the DG. The morphology and location of the BrdU/RCA-positive cells suggested that they were endothelial or microglial cells. BrdU/NeuN-positive cells were higher at P17 and P42 in the DG (Table 1).

### Hypoxia alone versus hypoxic-ischemic side differences

Table 2 shows the trends in the DG at the three sacrifice times for hypoxia alone and hypoxic-ischemic sides of the brain. BrdU-positive cells without co-labeling and BrdU/RCA-positive cells occur first after the H-I injury (significant increase at P10), followed by BrdU/NeuN-positive cells (significant increase at P42). Colabeling of BrdU and NeuN was confirmed by confocal microscopy (Figure 2). For BrdU/CNPase-positive cells the only significant difference was seen in the DG at 10 days, where the hypoxia alone side was significantly higher (7.0 ± 24.2 v. 0.1 ± 0.3, p < 0.0002). No significant differences were seen in the BrdU/GFAP-positive cells when the hypoxic-ischemic side was compared with the hypoxia alone side. Both GFAP positive and CNPase positive cells without BrdU staining were seen in appropriate areas.

## Discussion

The hippocampus has been the focus of much research involving H-I injury, both for its susceptibility to H-I injury and its regenerative capacity due to the adult neural stem cells found there. The bulk of this work has been compiled using adult models of H-I injury. In the neonatal mouse, the degree of damage to the hippocampus from this type of injury has been found to be variable, based on multiple factors, including the strain of mouse used [16]. In addition, the timing of the insult has been demonstrated to affect the severity of damage. The mouse hippocampus is remarkably resistant to H-I injury at 2–3 post-natal days but becomes progressively vulnerable, and by age 13 post-natal days hippocampal damage exceeds that of cortex [16]. Scheepens et al [17] found an increase in proliferation of hippocampal cells following global asphyxia in the newborn rat, much as we found in the model we used, but these investigators did not assess maturation of the proliferating cells into neurons and other cell types. In one study neonatal hypoxia alone triggered neurogenesis in a neonatal rat model [18]. The changes they noted were mainly in the CA1 region, as opposed to the DG, where we found the primary changes. Because the hippocampus is vulnerable to H-I damage and has the potential for neurogenesis, yet has not been extensively examined in a neonatal model, the hippocampus was the focus for this study.

We can summarize the regenerative process as follows: the initial phase is characterized by comparatively large numbers of incompletely differentiated cells, primarily in the DG (10 days of age or 3 days after injury), followed rapidly by the appearance of BrdU/RCA-positive cells, again mainly in the DG, and then by BrdU/NeuN-positive cells (putative neurons) developing only in the DG (at 42 days of age). There were minimal numbers of BrdU/GFAP-positive cells observed at all points.

Ischemia has been shown to increase neurogenesis in several adult animal models of brain ischemia. Following global cerebral ischemia in adult gerbils, neurogenesis has been shown to increase in the hippocampal SGZ, while newly born neurons were demonstrated in the CA1 pyramidal cell layer [8]. In another study of adult gerbils, after 10 minutes of bilateral carotid occlusion, newborn cells with a neuronal phenotype were first seen 26 days after injury [5]. In the adult rat, ischemia has been shown to increase the incorporation of BrdU-positive cells co-expressing neuronal markers in both the DG and the SVZ [7]. Furthermore, this effect was more prominent on the side of ischemic insult [7]. Similar studies in the adult rat demonstrate that up to 60 percent of the new cells found after this type of ischemic brain damage demonstrate neuronal characteristics. In adult mice, after transient forebrain ischemia, BrdU-positive cells have been demonstrated to significantly increase in the DG [4]. In view of the fact that we observed the BrdU/NeuN-positive cells to increase only in the DG, it seems probable that the regenerative process in neonates is similar to that in adult animals.

The data strongly suggested that the development of oligodendrocytes (BrdU/CNPase-positive cells) was impaired in the DG on the hypoxic-ischemic side of the brain, with significantly fewer of these cells observed in the hypoxic-ischemic side as compared to the hypoxia alone side at 10 days. This finding is in contradiction to findings reported by Zaidi et al [19], who demonstrated that neonatal H-I injury in rats led to increased numbers of oligodendrocytes ipsilateral to H-I injury. There are several possible explanations for this discrepancy. In our study, oligodendrocytes were found to be decreased on the side of the brain ipsilateral to carotid ligation at P10. In the Zaidi study, they were found to be increased at P35. It is possible that oligodendrocytes proliferate at the site of damage after P10. It is also possible that the oligodendrocytes are at the site of damage at P10, but are not mature enough to stain with CNPase. Lastly, the Zaidi group evaluated the SVZ, while we reported dual staining in the hippocampus. There could be regional differences between the two.

White matter damage in neonates is thought to be in part due to vulnerability of the immature oligodendrocyte to H-I damage [20]. In several neonatal animal models, oligodendrocytes have been demonstrated to be susceptible to H-I injury [21-24]. This effect seems to be dependent on both the degree of H-I damage [24] and the timing of the injury, with late oligodendrocyte progenitors being more susceptible than early oligodendrocyte precursors or more mature oligodendrocytes [25]. Our results were consistent with these reports. This window of vulnerability of the oligodendrocyte coincides with the human population at high risk for the development of PVL. This vulnerability of the immature oligodendrocytes might explain the paucity of new, mature oligodendrocytes in our model on the hypoxic-ischemic side [26].

We found a significant, early increase in BrdU-positive cells expressing RCA, an endothelial cell marker and microglial marker, on the side of the brain affected by hypoxic-ischemic injury. The mechanisms of angiogenesis in the brain after H-I injury are not well understood, and there is also little known about angiogenesis in neonatal animal models. Vascular endothelial growth factor (VEGF) is endothelial cell specific and has been implicated in hypoxia-mediated angiogenesis [27]. VEGF has also been demonstrated in microglial cells after cerebral infarct in adult rats [27]. In the rat brain, angiogenesis is not completed until about post-natal day 20 [28]. Therefore, it remains a possibility that the increase in cells co-labeling for BrdU and RCA after H-I injury in our study partly represents the normal brain maturation process. However, the increase of these cells on the hypoxic-ischemic side of the brain would imply that the injury led to this effect. Further studies will be needed to more accurately identify if there are changes in angiogenesis following H-I injury. The majority of these BrdU positive/RCA positive cells appeared morphologically to be activated microglia, which are thought to play a role in the damage to immature oligodendrocytes [20].

The present study did not demonstrate an increase in BrdU-positive cells co-labeling for an astrocyte marker, GFAP, after H-I injury. Astrocytes have been demonstrated to express apoptotic enzymes within hours after H-I injury in neonatal rats [29]. In neonatal piglets, GFAP-positive cell bodies were reduced by roughly 50% at 48 hours after H-I injury, but subsets of astrocytes were subsequently shown to proliferate later after the insult [30]. In adult rats, the number of astrocytes was shown to remain unchanged after traumatic brain injury in one study [31], while in another similar study, there was loss of GFAP reactivity in the ipsilateral CA3 region of the hippocampus 30 minutes after injury with progressive astrocyte loss over the next 24 hours [32].

As evidenced by the variability reported in the astrocytic response to H-I brain injury, the response of this cell in this environment needs to be better characterized. Because astrocytes help to maintain the extracellular milieu needed for neuronal formation and function, it is possible that their early loss after H-I injury may contribute to subsequent neuronal degeneration. Future studies in our neonatal mouse model could be geared towards better understanding the early response of astrocytes. Postponing evaluation until three days post-injury in this model likely resulted in underestimation of the astrocytes' role in this injury repair process. Chronic hypoxia has been demonstrated to induce astrocytes into more immature phenotypes, which do not express normal levels of GFAP [33]. The ability of acute hypoxia to cause this effect is not well understood. Therefore, it remains a possibility that evaluating for immature astrocytes in this model would have resulted in the visualization of more astrocytic cells.

Although the BrdU-positive cells in our study were co-labeled with stains identifying four separate types of cells, there were many BrdU-positive cells that were not identified by these four stains. The co-labeling studies we performed accounted for only about 20% of all the BrdU-positive cells. The BrdU labeling protocol we used consisted of BrdU injections over the course of the experiment, rather than a one time injection at the time of H-I injury, which resulted in the staining of more cells than would be found following a single injection. In this manner, we were able to evaluate how each cell type varied its number and location at different intervals after the injury. This provided information about the cellular response to hypoxic-ischemic brain injury in both the acute and long-term settings. The role and identity of the non-co-labeled cells remains unclear. It is probable that these were immature cell types, not yet expressing the markers for which we evaluated.

The validity of cells staining for BrdU representing new or dividing cells versus apoptotic or necrotic cells is also an issue to be considered. As a result, TUNEL staining was performed on several hippocampal sections. At P10, three days after H-I injury, TUNEL staining does reveal apoptotic and necrotic nuclei. However, by P42, this finding is virtually non-existent, suggesting that any BrdU-positive cells are present due to cell proliferation or growth. (See Figures 4 and 5.) Previous studies have indicated that neurons subjected to H-I injury do, indeed, incorporate BrdU, but that these injured neurons which undergo apoptosis have disappeared by 28 days [34].

A possible criticism of the present study is that we did not count the cells with stereological methods. As a result, our findings might be due to increased cell density due to surrounding tissue loss as opposed to an increase in the absolute number of new cells present. However, the cell counts were conducted in non-overlapping sections within a very short segment of the anterior hippocampus, in which the sections' thickness remained constant. No gross differences were observable in brain size in either the coronal or sagittal planes. Furthermore, hippocampal volume on both the hypoxia alone and hypoxic-ischemic sides of the brain did not vary noticeably histologically. Lastly, confocal imaging revealed cell density on each side of the brain to be roughly equivalent, although exact volumetric measurements can not be made without stereologic techniques.

## Conclusion

Neuronal and other cell regeneration was significantly increased in the DG on the side of the brain exposed to H-I injury, as opposed to hypoxic injury alone. This study addresses the neonatal mouse brain's ability to repair H-I damage and can serve as a platform to determine if interventions can be made to augment these inherent repair mechanisms. The rate of neuronal regeneration found in our study, while showing a significant increase in the DG, was not robust. Perhaps the stimulus was insufficient to result in a great degree of neurogenesis. The degree of brain injury was not stratified in this study. Neurogenesis might prove to be proportional to the degree of injury. It is also possible that this regeneration could increase further with time, but this is unlikely because new cell production was decreasing by the final time point after the injury in our study. Stem cell transplantation or manipulations of factors affecting intrinsic stem cell activity could be applied to this system in the future.

## Methods

### Animal procedures

**Figure 1:**
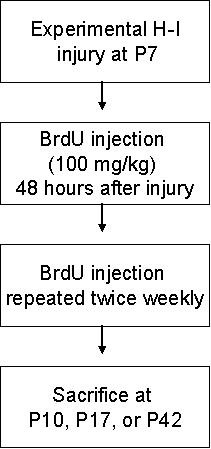
Schematic diagram showing BrdU injection schedule

This study was performed in accordance with the guidelines provided by the Laboratory Animal Studies Committee of the Veterans Administration Hospital in Augusta, GA. Under 2% isoflurane anesthesia C57 BL/6 mouse pups underwent permanent ligation of the left common carotid artery at post-natal day of life seven (P7). The pups were then placed with the dams for two hours prior to placement in an 8% oxygen chamber partially immersed in a water bath at 37° for 75 minutes. This procedure was described previously in the rat by Levine [12] and Rice *et al *[13], and then adapted to the mouse by Sheldon *et al *[14]. Within the literature, there is variation of the neonatal mouse H-I brain injury protocol. Our seventy-five minute hypoxia time was determined by exposing animals to varying intervals of hypoxia. In our laboratory, seventy-five minutes of hypoxia time resulted in reliable, reproducible brain injury, with minimal animal death. Any animal that died during surgery, while in the hypoxia chamber, or prior to sacrifice time was excluded. The time at which the animal would be sacrificed was determined prior to surgery. Forty-eight hours after the H-I injury, the pups were injected intraperitoneally with BrdU, at a dose of 100 mg/kg. This dose was administered twice weekly until the pups were sacrificed at 3, 10, or 35 days after the injury (see Figure 1) with 70 mg/kg of ketamine and 15 mg/kg of xylazine, and tissue fixation done by transcardiac perfusion with 0.9% saline, followed by 4% paraformaldehyde in PBS. BrdU injection was continued twice weekly after injury until the time of sacrifice in order to elucidate if the types of new cells found in the hippocampus varied at different time intervals after the injury. This protocol allowed evaluation of an accumulation of cells, reflecting both the acute and long-term cellular response to hypoxic and hypoxic-ischemic injury. Brains were removed and postfixed in 4% paraformaldehyde for 4 hours, cut into 3–5 mm sections, and postfixed in 4% paraformaldehyde for an additional 20 hours. Tissue was then dehydrated in an ethanol series, cleared in xylene, and infiltrated and embedded in PolyFin Embedding and Infiltration Wax (TBS Biomedical Sciences, Durham, NC). Paraffin embedded tissue was sectioned to 5 μ thickness and mounted on Fisher Superfrost Plus slides (Fisher Scientific).

### Immunohistochemistry and cell counting

Paraffin was removed from slides using xylene, followed by rehydration in an alcohol dilution series. Slides were soaked in 0.1% Triton-X 100 in PBS for ten minutes to increase permeability of fixed tissue, followed by rinsing in 1X PBS. Antigen retrieval was performed using a microwave method. Slides were incubated for twenty minutes after slow boiling for ten minutes and then rinsed in PBS and blocked using 2% normal calf serum for 20 minutes.

Sheep anti-BrdU antibody (Biodesign #M20105S), diluted 1:200 in PBS, was selected for the visualization of new cells. Co-labeling runs used mouse anti-NeuN (Chemicon #MAB377) 1:2000 for the visualization of neurons, rabbit anti-GFAP (DAKO # Z0334), diluted 1:300 in PBS for the visualization of astrocytes, biotinylated RCA (Vector #B-1085) diluted 1:300, for the visualization of endothelial cells and microglia cells, and CNPase (Sigma #C-5922), diluted 1:200, for the visualization of oligodendrocytes. All primary antibodies except NeuN were allowed to incubate at room temperature for one hour.

Primary NeuN antibody was allowed to incubate for thirty minutes at room temperature, and washed three times in a 1X PBS solution. Biotinylated anti-mouse antibody (Vector *Elite *ABC Kit #PK-6102), diluted 1:200 in 1.5% normal horse serum, was applied to each section and incubated for thirty minutes at room temperature. Slides were washed in a PBS series before applying Vector *Elite *ABC reagent and incubation for thirty minutes at room temperature. Sections were washed again in PBS and TSA amplification reagent (NEN #SAT700) with biotinylated tyramide 1:200 in amplification buffer was applied. Sections were incubated for eight minutes at room temperature.

Preliminary staining demonstrated that only NeuN antibody required amplification. All other antibodies were used in an indirect staining method, with a secondary, fluorescent antibody. Cy3 anti-Sheep (Jackson # 713-165-147), diluted 1:400, was used to visualize BrdU in all cases. FITC anti-rabbit (Jackson # 711-095-152), diluted 1:100, was used to visualize GFAP. Strepavidin FITC (Jackson #016-010-084), diluted 1:100, was used to visualize biotinylated RCA and NeuN. FITC anti-mouse (Jackson #715-095-151), diluted 1:100, was used to visualize CNPase.

To reduce background, slides were rinsed in 4 changes of PBS for 5 minutes each. Secondary antibodies were applied and incubated for 1 hour. Slides were rinsed in PBS, and immersed in Hoechst Stain for 8 minutes. Another rinse series in PBS was applied before coverslipping, using Vectashield Mounting medium. (Vector #H-1000)

**Figure 2:**
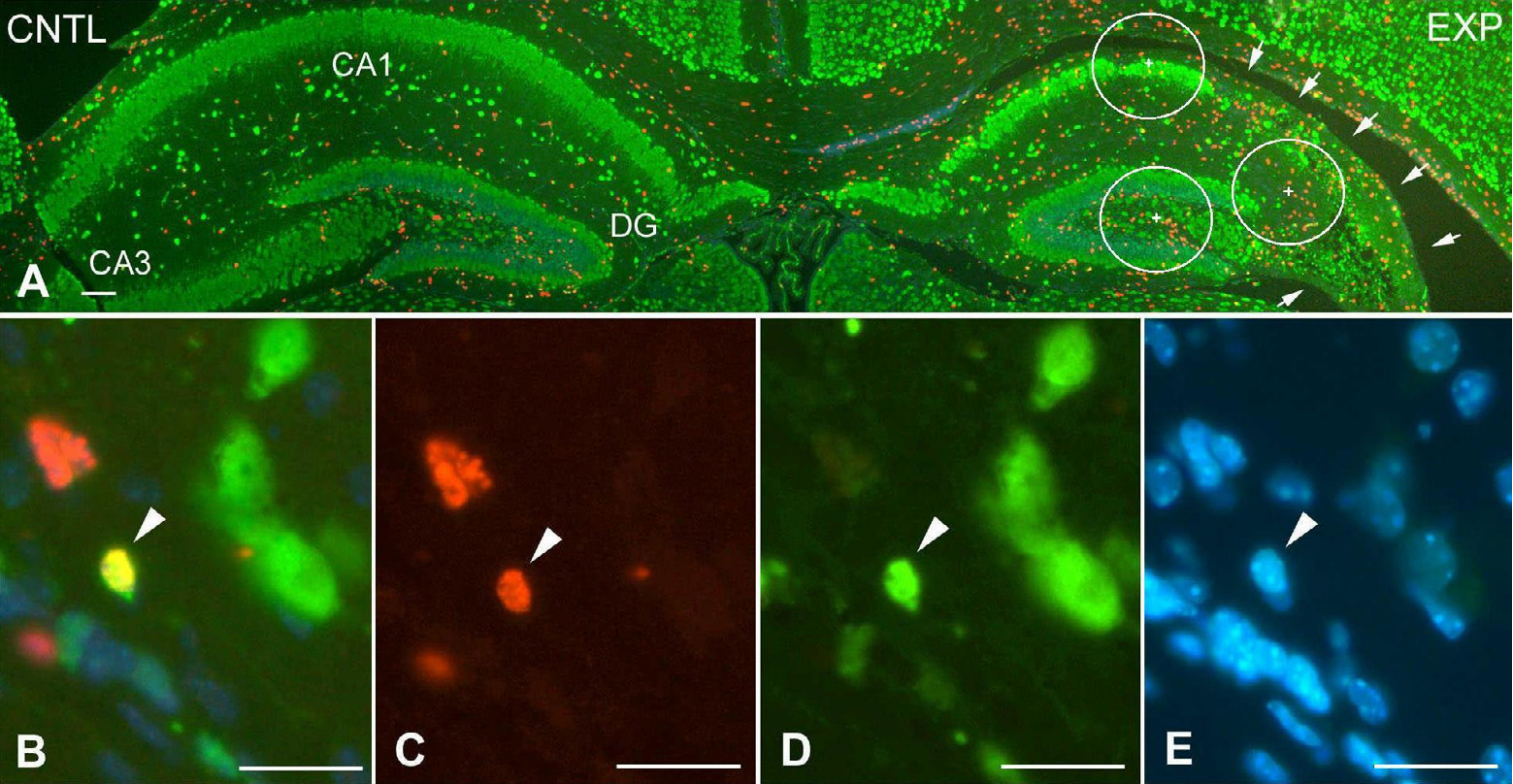
Hippocampal injury following hypoxic-ischemic injury in a P17 mouse. A – HI injury (small arrows), triple filter 100X; B – colocalization with NeuN and BrdU, triple filter, 400X; C – BrdU label alone, Cy3 filter, 400X; D – NeuN label alone, FITC filter, 400X; and E – Hoechst counterstain, DAPI filter, 400X. B through E shows the same field. The large arrows show a neuron with BrdU and NeuN staining, demonstrating that the cell has recently divided. The circles show the three areas where the counting was performed.

Cells were counted in 4–6 animals at each time interval. For each animal, the cells were counted from the injury area in the anterior hippocampus (equivalent adult mouse interaural coordinates 2.10 to 1.98 mm) [15] in CA1, CA3, and DG. (In Figure 2, the circles show the specific fields, CA1, CA3, and the DG, counted under one 40× magnification field in each section of brain. Corresponding areas of hippocampus were estimated in each section of brain when choosing the 40X field.) At least three brain sections from each animal for each stain type were counted. Therefore, each brain had three CA1, three CA3, and three DG 40X fields stained and counted with BrdU, one of the four co-labeling cell markers, and Hoescht stain. In each case, the same observer performed the cell counting in all brain sections. The counter could not be blinded due to the loss of normal architecture on the side of the brain consistent with H-I injury. A separate observer did count numerous sections in order to ensure consistency and accuracy. Each 40X field counted by separate individuals was found to have consistent data. Counting was completed by counting the total number of cells labeled for BrdU only and for BrdU co-labeled with one of the four the cell markers found in one field under 40× magnification in each of the three regions in the hippocampus, on both the hypoxia alone and hypoxic-ischemic side of the brain. Due to the large size of the hippocampus and the H-I injury, analysis was confined to one level of the brain cut through the middle of the injury. Any BrdU-only cells with abnormal morphology and any co-labeled cells were confirmed by corresponding Hoescht staining.

### Confocal imaging

**Figure 3:**
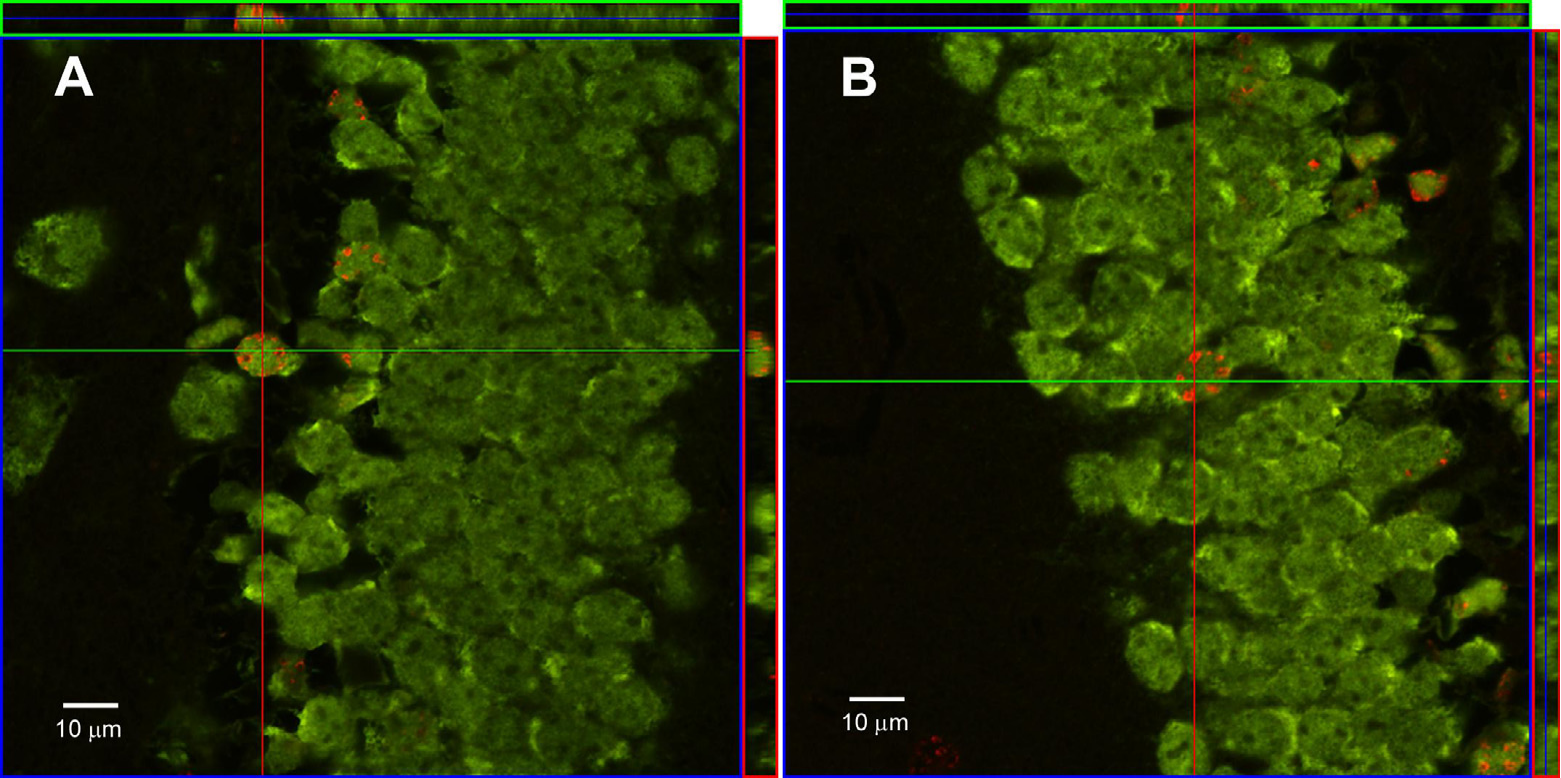
Three dimensional reconstructed confocal microscopy demonstrating colabeling BrdU positive and NeuN positive cell in granule cell layer of HI injured hippocampus (P42). Orthogonal view. The neurons are labeled with NeuN (green), and the BrdU positive cells are shown in red. The square is a the Z slice, the superior rectangle is the X slice, and the right rectangle of the Y slice. A and B show two different cells, both co-labeled with BrdU and NeuN.

**Figure 4:**
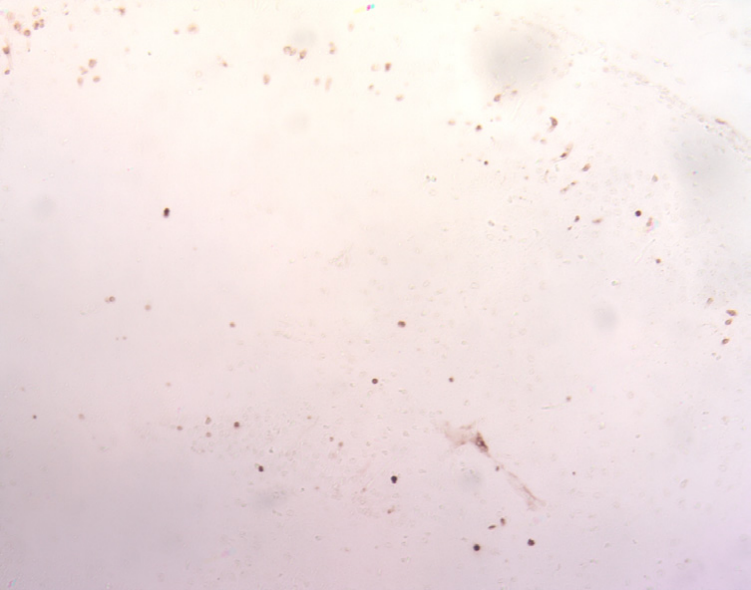
200× magnification of TUNEL staining developed with stable DAB in the injured hippocampus of a ten day old animal (P10), showing a small amount of label. Brown coloration indicates 3'OH DNA termini (apoptotic or necrotic cells).

**Figure 5:**
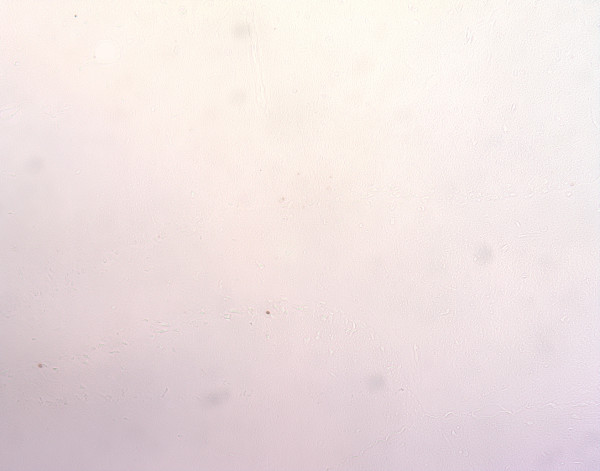
200× magnification of TUNEL staining developed with stable DAB in the injured hippocampus of a 42 day old animal (P42), showing no labeling of apoptotic or necrotic cells. Brown coloration indicates 3'OH DNA termini (apoptotic or necrotic cells).

To confirm dual labeling, confocal imaging was performed in selected cells with a Zeiss 510 laser scanning confocal microscope with the use of Zeiss software. The objective used was Zeiss63x C-Apochromat 1.2 NA. To excite the FITC fluorochrome (green), a 488-nm laser line generated by an argon laser was used, and for the Cy3 fluorochrome (red), a 543-nm laser line from a HeNe laser was used. Filter sets used were a bandpass 500- to 600- filter ("green" channel) and a long-pass 585- to 650-nm filter ("red" channel). We identified cells that were labeled for BrdU and the neuronal marker NeuN. These cells were then examined by confocal microscopy, and 1-μm step Z series were obtained. (See Figure 3.)

### TUNEL staining

Staining for the presence of apoptotic cells was completed using the ApopTag Peroxidase Kit (Chemicon #S7100), according to manufacturer recommendations. Therefore, after rehydration, the tissue was treated with Proteinase K (20 μg/mL) and incubated for 15 minutes. Endogenous peroxidases were quenched with hydrogen peroxide. Sections were then treated with equilibration buffer and TdT enzyme treatment. Next, the sections were stained with anti-dioxigenin peroxidase conjugate for 30 minutes, and the slides were developed with stable DAB.

### Statistical analyses

Nested, repeated measures analysis of variance was used to examine differences in number of cells between times of sacrifice (10, 17, or 42 days), side of brain (hypoxia alone or hypoxic-ischemic injury), region of the hippocampus (CA1, CA3, DG) and antibody type (CNPase, GFAP, NeuN, and RCA). A second analysis was performed using only BrdU-positive cells. Post hoc analyses were performed using the adjusted least square means and applying a Bonferroni correction to the overall alpha level. Statistical significance was assessed using an alpha level of 0.05. All analyses were performed using SAS 8.2.

## Abbreviations

hypoxic-ischemic – H-I, bromodeoxyuridine – BrdU, subventricular zone – SVZ, subgranular zone – SGZ, dentate gyrus – DG, glial fibrillary acidic protein – GFAP, 2'3'-cyclic nucleotide 3'-phosphodiesterase -CNPase, *Ricinus communis *agglutinin I –s RCA, neuronal nuclear marker – NeuN

## Figures and Tables

**Table 1:** Regional differences in injured side: Numbers of cells

Age at sacrifice: stain	CA1	CA3	DG
P10: BrdU	120.6 ± 39.7	109.7 ± 37.4	154.5 ± 59.6*
P10: BrdU/RCA	19.8 ± 12.2	15.1 ± 6.1	30.5 ± 17.8**
P10: BrdU/NeuN	0	0	0.2 ± 0.6
			
P17: BrdU	61.4 ± 21.7	49.6 ± 17.1	98.7 ± 26.2*
P17: BrdU/RCA	12.5 ± 4.1	6.2 ± 4.5	8.6 ± 3.8
P17: BrdU/NeuN	0.4 ± 0.7	0.3 ± 0.6	11.6 ± 4.4**
			
P42: BrdU	31.7 ± 19.4	35.0 ± 21.6	68.9 ± 23.4*
P42: BrdU/RCA	5.7 ± 5.8	2.7 ± 2.0	6.3 ± 4.5
P42: BrdU/NeuN	0	0.3 ± 0.8	15.0 ± 4.6**

*Significantly greater than CA1 or CA3, p < 0.0011

**Significantly greater than CA1 or CA3, p < 0.0002

**Table 2:** Injured versus non-injured dentate gyrus side differences: Numbers of cells

Stain: Side of brain	P10	P17	P42
BrdU			
Injured	154.5 ± 59.6*	98.7 ± 26.2	68.9 ± 23.4*
Non-injured	92.9 ± 32.7	86.7 ± 21.5	52.4 ± 17.1
BrdU/RCA			
Injured	30.5 ± 17.8**	8.6 ± 3.8**	6.3 ± 4.5
Non-injured	2.7 ± 2.6	2.4 ± 2.4	1.1 ± 1.1
BrdU/NeuN			
Injured	0.2 ± 0.6	11.6 ± 4.4	15.0 ± 4.6**
Non-injured	0.1 ± 0.3	6.6 ± 3.2	5.2 ± 1.6

*Significantly greater than control, p < 0.0011

**Significantly greater than control, p < 0.0002
